# On the Proximate Cause of Inflammation

**Published:** 1828-11

**Authors:** 


### INFLAMMATION.

*On the Proximate Cause of Inflammation.* By Dr. Bow.

With regard to the proximate cause of inflammation, I may say I am a believer in the doctrine which acknowledges diminished action in the capillaries. The objections to it, which by some are thought to be insurmountable, appear to me easy of explanation, and which never could have arisen had the advocates of the doctrine been a little less exclusive. Mere diminished action in the capillaries cannot account for all the symptoms which characterise inflammation; besides, what is there in this hypothesis to account for the diminished action itself?

Diminished action of the capillaries can only be the result of previous over-excitement, by which their contractility is exhausted, or the result of sudden abstraction of this power. We know it is not the consequence of the former, but in all likelihood it is of the latter.

You are aware I maintain that, if there be a determination of nervous influence to a part greater than natural, there will be a corresponding deficiency in its determination elsewhere. As soon as the remote cause of inflammation is applied, there is a transmission of nervous influence in excess to the part: this cause acts upon the sentient nerves; consequently to their extremities is this excess of nervous influence transmitted. The office of sentient nerves is neither that of conveying contractility to muscular fibre, nor of that modification of nervous influence which effects secretion; so that, although there be a greater than natural determination of influence to the part, there is neither an increase in the action of the capillaries, nor in the products of secretion. On the contrary, there is diminished action and defective secretion; for almost all the influence of the part is directed through the channel of sentient nerves. As soon as the capillaries are thus robbed of nervous influence, in which consists their contractility, they can no longer resist the influx of blood; and those of them whose office it was to carry colourless fluid become now distended with blood: hence the increased redness and swelling. This distention, in its turn, becomes an additional source of irritation, and thus, from a mere puncture, will inflammation spread around.

If this view of the proximate cause of inflammation be correct, it is sufficiently simple; and, with similar notions of nervous action, may we not attempt to explain many phenomena which yet puzzle the physiologist. The blush of shame or of modesty is caused by a sudden determination of nervous influence to the extremities of the sentient nerves of the face: they become, if I may so express myself, positively excited at the expense of those nerves in their vicinity whose functions are contractility and secretion: thus the capillaries, from the loss of their contractility, are suddenly distended; hence the phenomenon. As the sensation which created the blush subsides, the balance in the distribution of nervous influence is restored, and with it the proper functions of the part are renewed.

At the age of puberty, those changes in the body which have been attributed to sympathy may likewise be accounted for. At this age, genitalia evolvuntur, mammæ efflorescunt; yet this change does not result from increased action in the vascular construction of the parts, but rather from diminished contractility, following an increased determination of nervous influence to the sentient nerves, caused by the perception of sensations before unknown.

Many of the phenomena attending pregnancy may be referred to a partial loss of contractility, owing to the demand of nervous influence created by the new action of the uterus. The mammæ swell in consequence, and they do not return to their former size after parturition, because the nervous influence which before was transmitted to the uterus is now directed to the secretory nerves of the breast. When lactation ceases, however, then is the balance in the distribution of nervous influence restored; the vascular construction of the mammæ regains its contractility, and they diminish in size.

A thousand other phenomena may be explained in like manner, especially those observed in erectile tissues.

