# Errata

**Published:** 1828-12

**Authors:** 


ERRATA.—P. 402, 7th line from bottom, for *on* read *or*.
P. 403, 20th line from top, for *puncture* read *punctum*.

